# Tinea Unguium and Tinea Pedis and Their Correlation With Diabetes Mellitus in the General Population in the Hail Region, Saudi Arabia: A Cross-Sectional Study

**DOI:** 10.7759/cureus.40116

**Published:** 2023-06-08

**Authors:** Amany Khalifa, Ibrahim G Alreshidi, Lama A Alaradi, Yasmeen M Alrashidi

**Affiliations:** 1 General Medicine, University of Hail, Hail, SAU; 2 Dermatology, University of Hail College of Medicine, Hail, SAU; 3 College of Medicine, University of Hail, Hail, SAU

**Keywords:** ksa, hail, diabetes mellitus, tinea unguium, tinea pedis

## Abstract

Background

*Tinea pedis* or foot ringworm is an infection of the feet affecting the soles, interdigital clefts of toes, and nails, with a dermatophyte fungus. It is also called athlete’s foot. Onychomycosis of the nail is caused by dermatophytes called *Tinea unguium*. An abnormal nail not caused by a fungal infection is a type of dystrophic nail. Onychomycosis can infect both fingernails and toenails, but onychomycosis of the toenail is much more prevalent.

Aim

The study aimed to assess the knowledge, perception, and awareness among a sample from Ha’il City, Saudi Arabia, of the definitions, risk factors, symptoms, diagnosis, complications, and treatment of both *Tinea pedis *and *Tinea unguium*, along withtheir correlation with diabetic patients.

Material

A cross-sectional survey was distributed throughout Ha’il City. An online questionnaire was designed and distributed via various social media apps, which included questions concerning participants’ sociodemographic information, alongside questions regarding the risk factors, signs, symptoms, complications, and management of both *Tinea pedis *and *Tinea unguium.*

Methods

SPSS for Windows v22.0 (IBM Corp. Released 2013. IBM SPSS Statistics for Windows, Version 22.0. Armonk, NY: IBM Corp.) was used for statistical analysis.

Results

The overall awareness of the study’s participants about *Tinea Pedis *and* Tinea unguium* infection was low (34.82%).

## Introduction

Diabetes mellitus is a chronic metabolic disease, which has a steadily growing frequency [1,2]. Roughly 30% of diabetic patients present with skin lesions, and fungal skin infections are a notable example [3]. Research indicates chronic hyperglycemia in patients impacts cellular immunity and polymorphonuclear leukocytes, which in turn impairs phagocytic functions. The development of cutaneous fungal and other bacterial infections can then result in patients with foot conditions [4,5].

Fungal infections affecting the feet such as *Tinea pedis* and *Tinea unguium* are widespread among the global population [6]. With respect to *Tinea pedis*, or as it is more commonly known athlete’s foot, interdigital inflammation is the most reported clinical manifestation. It results in fissuring and maceration, primarily in the skin between the toes. Plantar athlete’s foot is characterized by hyperkeratosis and squamous plaques covering the heels, soles, and sides of the infected foot. In regard to inflammatory conditions, vesicles, pustules, and sometimes bullae appear on the sole [7].

*Tinea unguium* (or onychomycosis) is a dermatophyte infection impacting the nails. Onychomycosis appears with high frequency among older persons, with prevalence rates as high as 50% recorded in the 70+ category [8]. The most reported clinical subtype is distal lateral subungual onychomycosis. This is a brownish-yellowish discoloration linked with onycholysis and subungual hyperkeratosis. Research has found almost 50% of persons with toenail onychomycosis have concomitant fungal skin infections, of which *Tinea pedis* is the most common [9].

Multiple risk factors had been reported for *Tinea pedis* and *Tinea unguium*. These include age, male sex, climate (high temperature and humidity), footwear, exercise, use of public facilities, home infections, and incidence of certain diseases such as obesity, diabetes, and vascular disorders, along with bone and joint diseases [10-17]. In non-diabetic persons, *Tinea* infections are only mildly symptomatic. In those with diabetes, however, infected areas can provide entry points and fistulas, which in turn may result in serious bacterial infections. Impacted areas in patients display as pruritic, squamous, erythematous, and macerated. Moreover, vesicles and pustules may also form [18].

The study aimed to assess the knowledge, perception, and awareness among a sample from Ha’il City, Saudi Arabia, of the definitions, risk factors, symptoms, complications, diagnosis, and treatment of both *Tinea pedis *and *Tinea unguium*, alongside their correlation with diabetes mellitus.

## Materials and methods

This cross-sectional study was conducted on a population sample drawn from the Ha’il community in Saudi Arabia. Participant recruitment was done through a convenience sampling method, wherein an electronic questionnaire was distributed on several social media platforms. The aims and objectives of the study were presented at the start of the questionnaire, thereby enabling the participants to make an informed decision about participation. Participants included both Saudi and non-Saudis, men and women. All were aged 18 years or older and residents of the Ha’il region of Saudi Arabia. The sample size for the present study was 255, with different age groups and nationalities included.

Data collection

From October to December 2022, data were collected through a self-administered online questionnaire composed of two sections that measured several variables. The first section concerned the participants’ sociodemographic information (age, gender, nationality, place of residence, educational level, etc.). The second section comprised 21 questions about participants’ awareness of *Tinea pedis* and *Tinea unguium*, along with their correlation with diabetes. This included specific questions about diabetes, risk factors, symptoms, signs, complications, diagnosis, and treatment of fungal infections.

Data analysis

After the data were collected, it was then modified, coded, and entered into the statistical software SPSS for Windows v22.0 (IBM Corp. Released 2013. IBM SPSS Statistics for Windows, Version 22.0. Armonk, NY: IBM Corp.). All statistical analysis was done using SPSS.

Consent and ethical approval

The present study had been reviewed and approved by the Research Ethics Committee (REC) of the University of Ha’il, Saudi Arabia, along with the University President. The research project was numbered H-2022-355 and dated 31/10/2022. Participants took part on a voluntary and informed basis. No personal identifiers were collected.

## Results

Data were analyzed using SPSS version 22.0. The frequencies, percentage, and mean and standard deviation were determined to describe the awareness of *Tinea pedis* and *Tinea unguium *infection and how it related to the participants’ sociodemographic information. The Mann-Whitney and Kruskal-Wallis tests were used to assess the difference in the mean rank score of awareness in terms of socio-demographic factors, with a “p-value” of more than 0.05 considered statistically significant.

The normality test

A one-sample Kolmogorov-Smirnov test was used to measure the data distribution normality. This found that the variable was not normally distributed (p<0.05). Moreover, the distribution of demographic factors was found to be inequivalent. The non-parametric tests (Mann-Whitney and Kruskal-Wallis) were consequently applied.

As shown in Table 1, a total of 255 patients participated in the study. Regarding sex, they were 71.37% male and 28.63% female. Seventy point five nine percent (70.59%) were aged between 18 and 30 years old. The majority (95.29%) were Saudi. Seventy point two percent (70.20%) lived in Ha’il City while 29.80% lived in nearby Ha’il villages. Seventy six point zero eight percent (76.08%) had a bachelor’s degree. Eighty three point five three percent (83.53%) lived alone while the rest lived with others. Seventy one point three seven (71.37%) were single while 27.06% were married.

**Table 1:** Sociodemographic data (N=255)

Factor		N	%
Gender	Male	73	28.63
	Female	182	71.37
Age	18-30	180	70.59
	31-40	36	14.12
	41-50	31	12.16
	51-60	4	1.57
	+60	4	1.57
Nationality	Saudi	243	95.29
	Non-Saudi	12	4.71
Place of residence	In Ha’il	179	70.20
	In near Ha’il villages	76	29.80
Educational status	Middle school	6	2.35
	High school	46	18.04
	College	194	76.08
	Master’s & higher education	9	3.53
Living status	Living alone	213	83.53
	Living with others	42	16.47
Social status	Single	182	71.37
	Married	69	27.06
	Divorced	4	1.57

Table 2 presents the results of the assessment of participant awareness of *Tinea Pedis* and *Tinea unguium *infection. Ten point two percent (10.20%) were diagnosed with diabetes mellitus type 1, 4.31% with type 2. 5.88% were diagnosed with diabetes mellitus more than 10 years ago, 3.14% were diagnosed between six and 10 years ago or less than a year, while 1.96% were diagnosed with diabetes mellitus between one to five years ago. 42.75% had heard about athlete’s foot (*Tinea pedis*) or *Tinea unguium*, and 10.98% had family members who had been diagnosed with athlete’s foot (*Tinea pedis*). One point nine six percent (1.96%) had been diagnosed with it themselves while 0.78% said they and a family member had both had it. On the other hand, 9.80% had a family member who had been diagnosed with *Tinea unguium *while 3.14% had been diagnosed themselves.

**Table 2:** Awareness of Tinea pedis and Tinea unguium infection (N=255) Mean score of awareness = 3.83±2.07 out of 11 * Indicate a = risk factors/signs

Statement		N	%
Have you been diagnosed with diabetes mellitus?	Yes, type 1	26	10.20
	Yes, type 2	11	4.31
	No	218	85.49
Since when have you been diagnosed with diabetes mellitus?	Less than a year	8	3.14
	Since 1-5 years ago	5	1.96
	Since 6-10 years ago	8	3.14
	More than 10 years ago	15	5.88
	Never been diagnosed	218	85.49
Have you heard about athlete’s foot (Tinea pedis) or Tinea unguium before?	Yes*	109	42.75
	No	146	57.25
Have you or a family member been diagnosed with athlete’s foot (Tinea pedis)?	Yes, only me	5	1.96
	Yes, family member	28	10.98
	Yes, a family member and me	2	0.78
	No	220	86.27
Have you or a family member been diagnosed with Tinea unguium?	Yes, only me	8	3.14
	Yes, family member	25	9.80
	No	222	87.06
Do you do sports?	Yes, football	10	3.92
	Yes, basketball	8	3.14
	Yes, swimming	11	4.31
	Yes, walking	119	46.67
	No	60	23.53
	Other	47	18.43
How long do you wear tight or closed shoes every day?	Less than 2 hours daily	41	16.08
	2- 4 hours daily*	57	22.35
	4 – 8 hours daily*	122	47.84
	More than 8 hours daily*	33	12.94
	I don’t wear them	2	0.78
Do you have any pets at home?	Yes*	51	20.00
	No	204	80.00
Have you visited a beauty salon to get your nails done (pedicure and manicure)?	Yes*	96	37.65
	No	159	62.35
Have you noticed any redness, itchiness, dryness, scaling, and fissuring of skin between your toes?	Yes*	47	18.43
	No	208	81.57
Have you noticed any foul smell or changes in the shape and color of your hand nails?	Yes*	24	9.41
	No	231	90.59
Do you think that wearing closed shoes for a long time increases the risk of Tinea pedis?	Yes*	128	50.20
	No	20	7.84
	I don't know	107	41.96
Have you used any of the creams or medications to treat fungal infections? Please choose the cream or medication you used.	Lamisil spray	9	3.53
	Lamisil cream	8	3.14
	Canesten cream	4	1.57
	Daktarin cream	11	4.31
	Nizoral cream	8	3.14
	No, I haven’t	238	93.33
Have you noticed bubbles (blisters) between your toes?	Yes*	35	13.73
	No	220	86.27
Have you used any oral anti-fungal medications?	Yes, treating nail fungal infection	1	0.39
	Yes, treating foot fungal infection	8	3.14
	No, I haven’t used them	247	96.86
Do you use any drugs that lower your immunity (e.g. cortisone, chemotherapy, biological drugs, etc.)?	Yes*	9	3.53
	No	246	96.47
Have you been diagnosed with peripheral arterial disease (PAD) before?	Yes*	4	1.57
	No	251	98.43
If you have diabetes mellitus, what is your average blood glucose level (hbA1c) while being diagnosed with Tinea pedis or Tinea unguium?	6.5% or less	4	1.57
	(6.6%–8%)*	11	4.31
	(8.1%–10%)*	32	12.55
	10.1% or more*	1	0.39
	Never been diagnosed with Tinea Pedis or Tinea Ungums	207	81.18

Concerning sporting activities, 46.67% had practiced walking. Football, basketball, and swimming scored less than 5% each. Eighteen point four three percent (18.43%) of participants did other sports. Forty-seven point eight four (47.84%) wore tight or closed shoes for four to eight hours daily while 22.35% wore them for two point four hours daily and 12.94% for more than eight hours daily. Twenty percent (20%) of the present study participants owned pets. Thirty-seven point six five (37.65%) had visited a beauty salon to get nails done (pedicure and manicure). Eighteen point four three percent (18.43%) noticed redness, itchiness, dryness, scaling, and fissuring of the skin between the toes, and 9.41% noticed a foul smell or changes in the shape and color of hand nails. Thirteen point seven three percent (13.73%) noticed bubbles (blisters) between the toes while 50.20% thought that wearing closed shoes for a long time increases the risk of getting *Tinea pedis*.

Using creams or sprays (Lamisil, Canesten, Daktarin, and Nizoral) to treat fungal infection did not exceed 5% each. Three point one four percent (3.14%) had received treatment for a foot fungal infection while only 0.39% received for a foot nail infection. Three point five three percent (3.53%) used drugs that lower immunity such as cortisone, chemotherapy, and biological drugs. One point five seven percent (1.57%) had been previously diagnosed with peripheral arterial disease (PAD). Twelve point five five percent (12.55%) reported they had a blood glucose level (HBA1c) of between 8.1% and 10% when diagnosed with *Tinea pedis* or *Tinea unguium *while 4.31% had a level between 6.6% and 8%. The mean score for participant awareness was 3.83±2.07 out of 11/low level, and the scoring of awareness was calculated using the Mann-Whitney and Kruskal-Wallis tests.

As shown in Table 3, the Mann-Whitney and Kruskal-Wallis tests were applied to present the differences in participants’ awareness with respect to the socio-demographic factors. There were significant differences in participants' awareness with regard to gender (p<0.05=0.041), with higher scores for females (mean rank=133.9) as compared with males (mean rank=113.28).

There were significant differences in participants’ awareness with regard to age (p<0.01=0.041), with the highest scores in the 18-30 bracket (mean rank=137.28) while those aged more than 50 had the lowest mean score. There were significant differences in participants' awareness in terms of social status (p<0.05=0.02), too. Single participants scored highest (mean rank=108.41) while divorced participants had the lowest mean rank.

Nationality, place of living, education status, and living status were found to have insignificant relationships with participant awareness (p>0.05).

**Table 3:** Differences in participants' awareness in relation to socio-demographic factors (N=255) *≤0.05; **≤0.01; *** ≤0.001

Factor		Mean rank	Statistic	P value
Gender	Male	133.9	U=5568.5*	0.04
	Female	113.28		
Age	18-30	137.28	X^2^=18.07**	0.002
	31-40	96.65		
	41-50	129.61		
	51-60	66.13		
	+60	42.13		
Nationality	Saudi	128.07	U=1442	0.95
	Non-Saudi	126.67		
Place of residence	In Ha’il	133.20	U=5871.5	0.08
	In near Ha’il villages	115.76		
Educational status	Middle school	108.67	X^2^=1.33	0.72
	High school	135.16		
	College	127.66		
	Master’s & higher education	111.67		
Living status	Living alone	127.33	U=4331	0.74
	Living with others	131.38		
Social status	Single	136.2	X^2^=8.22*	0.02
	Married	108.41		
	Divorced	93.13		

As shown in Table 4, the Mann-Whitney and Kruskal-Wallis tests were applied to present the differences in the awareness of *Tinea pedis* and *Tinea unguium* infection in terms of diabetes mellitus. Their data showed insignificant differences in the awareness of *Tinea Pedis* and *Tinea unguium* infection in terms of diabetes mellitus (p>0.05).

**Table 4:** Differences in the awareness of Tinea pedis and Tinea unguium infection in terms of diabetes mellitus (N=255) *≤0.05; **≤0.01; *** ≤0.001

Factor		Mean rank	Statistic	P value
diabetes mellitus	Yes, type 1	145.56	X^2^=2.03*	0.36
	Yes, type 2	138.55		
	No	125.37		

## Discussion

Cutaneous dermatophytosis is increasingly prevalent around the world, above all in the tropics. However, it has not received the level of research interest it demands. It is necessary to go back to the 2000s to find guidelines from the American Academy of Dermatology on the management of *Tinea corporis* and *Tinea* *cruris* [19]. More recent guidance from the British Association of Dermatology mainly looked at *Tinea capitis* and *Tinea unguium*, with little focus on *Tinea corporis/cruris* [20-22]. Updated Cochrane reviews concerning the use of topical therapies in cases of *Tinea corporis*, *cruris*, and *pedis*, alongside several concerning oral therapies, have taken a step toward filling this research gap. However, systematic clinical trials, evidence-based national/international guidelines, along with recommendations about what dose and duration of systemic antifungals should be used in cases of *Tinea corporis/cruris*, are still areas that are in urgent need of address [23-25].

Thus, to gain more information about foot and nail mycosis in Saudi society, the present study was carried out to assess the knowledge, perception, complications, and awareness of *Tinea pedis* and *Tinea unguium* in Ha’il City, KSA, along with their correlation with diabetes mellitus diseases. In the present study, there were significant differences in participants' awareness as regards gender (p<0.05=0.041), with more females (71%) than males (28%) in knowledge about *Tinea pedi*s and *Tinea unguium*. Similarly, Al Arawi et al. also reported that female participants in their study had significantly higher knowledge than males regarding diabetes and its complications (p=0.028) [26].

The present study also found significant differences in participants’ awareness in regard to age (p<0.01=0.041), with the 18-30 bracket demonstrating the highest awareness (70.59%) while those aged more than 50 (1.57%) had the lowest mean rank. There were also significant differences in participants’ awareness with respect to social status (p<0.05=0.02), with single (71.37%) participants having the highest and divorced participants with the lowest mean rank (1.57%). In a previous study, Toukabri et al. found data regarding foot mycoses frequency per age group showed those between 41 and 50 years had the highest infection rates (23.1%), followed by the 51 to 60 cohort (21.9%); however, the differences were not found to be statistically significant (p=0.0658 and p=0.71, respectively) [27].

On the other hand, the present research found that nationality, place of residence, education status, and living status had insignificant relationships with participant awareness (p>0.05). Forty-two point seven five percent (42.75%) had heard about athlete’s foot (*Tinea pedis*) or *Tinea unguium*, and 10.98% of family members had been diagnosed with athlete’s foot (*Tinea pedis*) while only 1.96% got the infection, and 0.78% both had it themselves as well as having a family member with it. Moreover, 9.80% of participants had a family member who had been diagnosed with *Tinea unguium* while 3.14% of the participants had been diagnosed themselves. In contrast to a previous study in Saudi Arabia, it was found that educational status had a significant association with the importance of foot care in DM (p=0.037) [26].

As regards the risk factors of mycosis, the findings of the present study were that 46.67% had practiced walking while football, basketball, and swimming were each done by less than 5% of participants, and 18.43% did other sports. Forty-seven point eight four percent (47.84%) wore tight or closed shoes for four and eight hours daily while 22.35% wore them for two to four hours daily and 12.94% for more than eight hours daily. Twenty percent (20%) of the present study participants owned pets, and 37.65% had visited a beauty salon to get their nails done (pedicure and manicure). Fifty point two percent (50.20%) thought that wearing closed shoes for a long time increases the risk of contracting *Tinea pedis*.

Toukabri et al. also examined possible risk factors [27]. Their research reported that participants practiced ritual ablutions (56.6%), followed by communal showers (50.5%) and a family history of foot mycoses (28.6%). However, there was no statistically significant association between these factors and foot infection (p=0.41, 0.631, and 0.246, respectively). A significant association was found between foot mycoses and nail trauma (26.5%; p=0.019), wearing used shoes (26.3%; p=0.001), antifungal drugs (25.7%; p=0.013), physical activities (14.7%; p=0.049), occlusive shoes (13.2%; p=0.008), swimming pools (8.09%; p=0.045), attending thermal station (8.3%; p=0.021), pedicure (14.1%; p=0.006), associated fingernails onychomycosis (7.5%; p=0.010), and taking immunosuppressive drugs (5.4%; p=0.018). Moreover, Sasagawa found there to be a significantly higher ratio of subjects who wore high‐temperature/high‐humidity footwear with respect to both *Tinea pedis* and fungal foot disease since the internal footwear environment was liable to become humid [28].

In the present study, regarding the clinical picture, 18.43% noticed redness, itchiness, dryness, scaling, and fissuring of skin between toes. 9.41% noticed a foul smell or changes in the shape and color of hand nails while 13.73% noticed bubbles (blisters) between the toes. Three point one four percent (3.14%) had treated their foot fungal infection. Three point five three percent (3.53%) had a history of using drugs that lower immunity such as cortisone, chemotherapy, and biological drugs. One point five seven percent (1.57%) had been previously diagnosed with peripheral arterial disease (PAD). Moreover, 10.20% had been diagnosed with diabetes mellitus type 1 while 4.315% had type 2. In addition, 5.88% were diagnosed with diabetes mellitus more than 10 years ago, 3.14% were diagnosed six to 10 years ago or less than a year ago, and 1.96% were diagnosed with diabetes mellitus between one to five years ago. Twelve point five five percent (12.55%) reported that had a high blood glucose level (HBA1c) (i.e. 8.1-10%) when diagnosed with *Tinea pedis* or *Tinea unguium* for 8.1%-10%, followed by 4.31% who had a level of 6.6-8%. However, there were insignificant differences in the awareness of *Tinea pedis* and *Tinea *unguium infection in patients with diabetes mellitus (p>0.05).

Similarly, Akkus et al. reported that there was no relationship found between the mean duration of diabetes, professions, treatment method, presence of co-morbid diseases, and native positivity and mycosis [29]. They also did find not any relationship between the presence of peripheral neuropathy and the incidence of *Tinea pedis*. Moreover, Toukabri et al. did not find a significant association between foot mycoses and diabetes, vascular disease, psoriasis, fungal infection of the skin, dermatological pathology, smoking, obesity, walking barefoot, and the application of henna (p>0.05) [27]. In contrast, in a previous study on the Saudi population, Mujammami et al. reported that university-educated healthcare workers had a high awareness level about diabetes complications, including feet problems, at significantly higher rates than university-educated nonhealthcare workers, with a p<0.05 significance level [30].

In the present study, the use of creams or sprays (Lamisil, Canesten, Daktarin, and Nizoral) to treat fungal infection did not exceed 5% for any one cream or spray. Foley et al. stated that an Efinaconazole 10% topical solution was the best to achieve a complete cure (i.e. normal‐looking nail coupled with fungus elimination, determined using laboratory methods), followed by a Tavaborole 5% solution [31].

The current study has made available important information for health practitioners, however, it also has some limitations. These include its cross-sectional design and data collection through self-reported measures, along with the absence of some variables such as management and outcomes. Therefore, studies supplemented with clinical assessment will be of value, in addition to patient-reported outcomes.

## Conclusions

In the present study, the overall awareness of participants about *Tinea pedis* and *Tinea *unguium infection was low, with a score of 34.82%. There were significant differences in participants’ awareness with regard to gender (p<0.05=0.041), with females (71%) scoring higher compared with males (28%). There were also significant differences in participants’ awareness with respect to age (p<0.01=0.041), with those aged 18-30 scoring highest (70.59%) while persons aged more than 50 (1.57%) had the lowest mean ranking. There were also significant differences in participants’ awareness in terms of social status (p<0.05=0.02), with single persons scoring highest (71.37%) while the divorced had the lowest mean rank (1.57%).

Nationality, place of residence, education status, and living status had an insignificant relationship with participants' awareness (p>0.05). Moreover, although only 10.20% of study participants were diagnosed with diabetes mellitus type 1 and 4.32% with type 2, there were insignificant differences in the awareness of *Tinea pedis* and *Tinea *unguium infection in patients with diabetes mellitus (p>0.05). Thus, this low level of awareness about foot and nail mycosis in the Ha’il region necessitates more efforts in providing health education about mycosis via social media, hospitals, and health centers, with a particular focus on patients at high risk of infection.
